# Therapeutic Potential of *Ishige okamurae* Yendo as a Multi-Target Inhibitor Against Dementia Symptoms

**DOI:** 10.3390/life15111699

**Published:** 2025-11-01

**Authors:** Oh Yun Kwon, Seung Ho Lee

**Affiliations:** 1 Department of Nano-Bioengineering, Incheon National University, 119 Academy-ro, Yeonsu-gu, Incheon 406-772, Republic of Korea; ohyun1220@naver.com; 2 Institute of New-Drug Development, Incheon National University, 119 Academy-ro, Yeonsu-gu, Incheon 406-772, Republic of Korea

**Keywords:** *Ishige okamurae*, dementia, Alzheimer’s disease, glutamate excitotoxicity, neuroinflammation, multi-target drugs, natural agents

## Abstract

*Ishige okamurae* Yendo (*I. okamurae*) is a brown macroalga with diverse biological activities. Recently, its ameliorative effects against dementia progression have been demonstrated in various in vitro and in vivo models of Alzheimer’s disease (AD), glutamate excitotoxicity, and bacterial-driven neuroinflammation. *I. okamurae* extract (IOE) inhibited AD progression by regulating amyloid beta–induced neuronal death and cognitive impairments. Moreover, IOE attenuated glutamate-induced neurodegeneration by modulating the mitogen-activated protein kinases/Nrf2/heme oxygenase-1 signaling pathway. Furthermore, IOE effectively suppressed lipopolysaccharide-mediated neuroinflammation and memory deficits by downregulating the Toll-like receptor 4/MyD88-dependent signaling pathway. Collectively, these findings highlight the potential of IOE as a natural multi-target, anti-dementia agent. In this review, we summarize the neuroprotective and cognition-enhancing properties of IOE, discuss the molecular mechanisms underlying its action, and consider the advantages of *I. okamurae* as a promising natural resource for effective therapeutic developments that are capable of targeting multiple pathogenic causes of dementia.

## 1. Introduction

Dementia is a syndrome characterized by a decline in cognitive function that interferes with normal daily life. Patients with dementia generally exhibit progressive impairments in memory, speech, and judgment that often lead to serious social and domestic problems. Consequently, specialized care is typically required at the later stages of life [1,2]. Dementia is not considered a single disease; it can be caused by a variety of neurological disorders and medical conditions. For example, dementia symptoms may result from neurodegeneration associated with Alzheimer’s disease (AD), Parkinson’s disease (PD), and amyotrophic lateral sclerosis (ALS) [3,4]. In addition, several medical conditions, including cerebrovascular disease, traumatic brain injury, and brain tumors, are recognized as major causes of dementia [5,6,7].

As human longevity increases, the prevalence of dementia also increases. The number of individuals living with dementia worldwide is projected to reach approximately 153 million by 2050 [8]. Although a few anti-AD drugs designed to neutralize amyloid plaques have been approved by the U.S. Food and Drug Administration (FDA), effective therapeutics that can address the diverse causes of dementia remain lacking [9,10]. Substantial efforts are underway to develop anti-dementia therapies, and several candidates are in preclinical stages, providing hope to affected individuals [11,12].

One strategy for anti-dementia drug development is to directly interfere with the pathological inducers of dementia. Antibodies targeting amyloid plaques and phosphorylated tau proteins have been widely explored [13,14]. In addition, various chemical or peptide-based antagonists of dementia-related molecules, such as N-methyl-D-aspartate receptors (NMDARs) and Toll-like receptors (TLRs), which mediate dementia-related signaling, have been investigated [15,16]. Another emerging approach involves the use of stem cells to replace or repair damaged neuronal tissue. Stem cell transplantation modulates the brain microenvironment by secreting neurotrophic factors and attenuating neuroinflammation, both of which are important for dementia progression. However, despite promising results in animal studies and preclinical trials, stem cell therapy carries risks including immune rejection and tumor formation due to abnormal differentiation. Moreover, human-derived stem cells pose ethical and regulatory issues [17,18,19]. Collectively, considering that dementia can be induced by multiple pathogenic factors, therapeutic agents targeting a single cause may have limited applicability to a wide range of patients. Therefore, the development of multi-target agents capable of modulating the diverse pathological mechanisms underlying dementia would offer an effective strategy for treating dementia.

Natural agents are attractive resources for the development of antidementia drugs because they contain diverse bioactive compounds with vast structural diversity, potentially producing synergistic effects on therapeutic efficacy. They may also contain unique chemical constituents that can serve as lead compounds for novel therapeutics [20]. In addition, natural agents are generally considered safer than newly synthesized chemicals or peptides [21]. Although the identification of effective natural agents requires extensive and time-consuming screening and validation, a successful discovery may offer unexpected opportunities for the development of new therapeutics.

*Ishige okamurae* Yendo (*I. okamurae*), a brown macroalga with thick cortical layers, narrow fronds, and acute apices, belongs to the Ishigeaceae family and is widely distributed on rocks in the intertidal zones of East Asia [22]. *I. okamurae* is consumed as a traditional food owing to its high nutritional content, which includes vitamins and minerals [23]. Macroalgae have evolved to produce diverse metabolites that enable them to survive under stressful conditions such as strong sunlight and temperature fluctuations. More than 3000 metabolites have been identified in macroalgae, and their types and quantities vary among species [24,25]. This metabolic diversity makes macroalgae attractive candidates for the development of safe and effective drugs against intractable diseases. *I. okamurae* exhibits anti-inflammatory, hypoglycemic, antiviral, anti-glycation, and renal protective activities [26,27,28]. Moreover, the antidementia properties of *I. okamurae* have been evaluated in three disease models: AD [29], glutamate excitotoxicity [30], and bacteria-driven neuroinflammation [31]. *I. okamurae* extract (IOE) exhibits potent inhibitory activity against amyloid-beta peptide–induced neuronal death and cognitive deficits. In addition, IOE treatment effectively attenuated glutamate-induced neuronal damage and memory loss. Furthermore, IOE administration markedly suppressed lipopolysaccharide (LPS)-stimulated inflammatory responses in neuronal cells and the mouse brain. Notably, IOE modulated distinct molecular targets to exert its antidementia effects in three different dementia models, indicating its potential as a therapeutic candidate capable of regulating multiple pathogenic mechanisms underlying dementia symptoms.

Despite these promising findings, no comprehensive review has summarized the anti-dementia properties of IOE. Therefore, the present review aimed to provide an overview of the therapeutic efficacy of *I. okamurae* demonstrated in three representative dementia models and to discuss its potential as a multi-target inhibitor against dementia symptoms in comparison with other natural agents derived from marine algae.

## 2. Ameliorating *I. okamurae* Activity in Amyloid Beta-Mediated AD

AD is a neurodegenerative disorder that induces symptoms of dementia, such as neuronal loss and cognitive deficits. Although numerous studies have investigated the etiology of AD, its precise mechanisms remain unclear. Among the proposed causes, several risk factors such as oxidative stress, neuroinflammation, acetylcholine deficiency, neurofibrillary tangles (NFTs) formed by hyperphosphorylated tau protein, and environmental factors have been suggested. However, abnormal amyloid beta (Aβ) plaque accumulation in the brain is widely considered a major trigger of AD [32]. Substantial increases in Aβ plaques have been observed in the brains of patients with AD, and these pathophysiological changes ultimately lead to neuronal death, directly linked with hallmark symptoms, including memory loss and cognitive impairment [33]. Numerous in vitro and in vivo studies have further demonstrated the severe toxicity of Aβ, showing that it activates apoptotic signaling and inflammatory responses in neurons [34,35,36]. Therefore, strategies that attenuate Aβ formation and promote Aβ clearance are regarded as promising disease-modifying therapies (DMTs). In fact, anti-AD drugs targeting Aβ have shown positive effects in alleviating AD symptoms in clinical studies [37]. Recently, antibody-based agents, such as lecanemab and aducanumab, which directly target Aβ, were approved by the U.S. Food and Drug Administration (FDA) [12]. However, although these monoclonal antibodies enhanced Aβ clearance and attenuated cognitive decline in patients with AD, they were also associated with amyloid-related imaging abnormalities (ARIA), raising concerns regarding their clinical value [38]. Thus, developing safe and effective agents capable of ameliorating Aβ-mediated cellular toxicity remains urgently needed.

The ameliorative efficacy of ethanol-extracted IOE against Aβ-induced AD progression was evaluated by Kwon and Lee [29]. In vivo studies using an AD mouse model constructed by intracerebroventricular (ICV) injection of Aβ showed that oral IOE administration significantly restored both short- and long-term memory function. Moreover, IOE markedly reduced neuronal death in the hippocampal region and de-creased Aβ plaque burden in the brains of treated mice, indicating its ability to inhibit Aβ plaque formation, a major contributor to AD progression (Table 1).

The effect of IOE on AD biomarkers was also examined. Expression of cleaved caspase-3, a key regulator of apoptosis, was significantly normalized in the brains of IOE-treated mice compared with that in the AD controls (ICV-injected with Aβ). In addition, IOE suppressed the expression of inflammation-related biomarkers such as COX-2 and iNOS. Collectively, these in vivo findings suggest that IOE exhibits sufficient activity to inhibit Aβ aggregation and thereby prevent Aβ-mediated neuronal toxicity (Table 1).

These in vivo results were further supported by experiments using PC-12 neuronal cells. IOE effectively prevented apoptosis induced by ectopic Aβ treatment and restored the expression of Aβ-mediated apoptosis- and inflammation-related genes. Furthermore, both in vivo and in vitro studies have identified the mitogen-activated protein kinases (MAPKs) pathway as a key signaling cascade involved in the anti-AD effects of IOE. IOE treatment restored the abnormal Aβ-induced activation of MAPKs in mouse brains and PC-12 cells.

**Figure 1:**
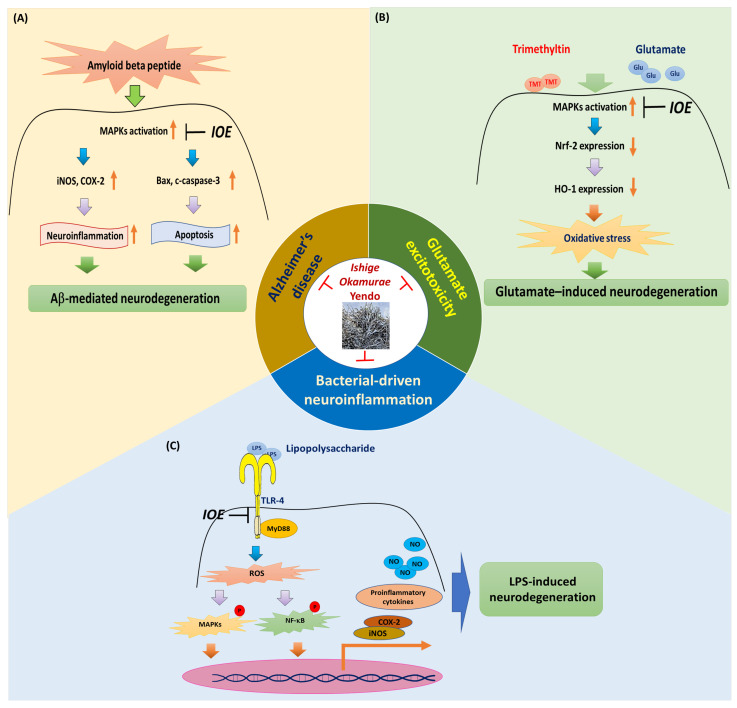
Anti-dementia properties of *I. okamurae*. *I. okamurae* extract (IOE) was proven to inhibit the progress of Alzheimer’s disease (**A**), glutamate excitotoxicity (**B**) and bacterial-driven neuroinflammation (**C**). Figure was prepared using Microsoft PowerPoint. Each schematic illustration (**A**–**C**) was adapted and modified from original publications of Kwon and Lee (2020) in Molecular Nutrition & Food Research [29], Kwon and Lee (2021) in Antioxidants [30], Kwon and Lee (2023) in Antioxidants [31] licensed under a Creative Commons CC BY license, respectively.

Aβ-mediated neuronal death is a critical step in AD progression. MAPKs are activated upstream to enhance the Aβ-induced expression of inflammation- and apoptosis-related genes. Therefore, inhibiting MAPK overactivation may hinder AD progression even in the presence of Aβ-induced neuronal stress. Preventing Aβ-induced MAPK overactivation could thus represent a promising anti-AD therapeutic approach. Since IOE effectively inhibited Aβ-induced MAPK activation in PC12 cells and mouse brain, the anti-AD effects of IOE are likely initiated by suppressing Aβ-mediated MAPK overactivation (Figure 1A, Table 1). In addition, the inhibitory effects of IOE on memory impairment observed in AD-model mice suggest that potential therapeutic compounds may be present in IOE and that these bioactive components are capable of penetrating the blood–brain barrier (BBB). Although diphlorethohydroxycarmalol (DPHC) has been identified as a single analytical component of IOE, further identification of functional constituents capable of attenuating AD progression would provide stronger evidence supporting the therapeutic potential of IOE in ameliorating Aβ-mediated pathology in AD.

## 3. Ameliorating *I. okamurae* Activity Against Glutamate Excitotoxicity

Glutamate is the most abundant neurotransmitter in the brain and plays an essential role in the transmission of signals between neurons. However, under pathological conditions, excessive accumulation of glutamate in the synaptic cleft can overstimulate glutamate receptors, particularly N-methyl-d-aspartate receptors (NMDARs), leading to intracellular Ca^2+^ overload [39,40]. Glutamate-induced Ca^2+^ accumulation triggers reactive oxygen species (ROS) generation, disrupts mitochondrial function, and activates apoptotic pathways, ultimately causing neuronal death [41]. This series of events, known as glutamate excitotoxicity, is a crucial step in the pathogenesis of several neurodegenerative disorders, including AD and PD [42,43].

Glutamate excitotoxicity is closely associated with AD pathology, as Aβ can directly activate NMDARs, resulting in increased Ca^2+^ influx [44]. Indeed, memantine, an FDA-approved NMDAR antagonist, has shown clinical efficacy in improving cognitive function and behavior in patients with moderate-to-severe AD [45]. Therefore, each step in glutamate excitotoxicity represents a potential therapeutic target for alleviating AD progression, particularly cognitive dysfunction, which is a hallmark of dementia.

Mitochondrial dysfunction induced by Ca^2+^ accumulation during glutamate excitotoxicity, increases oxidative stress and plays a crucial role in neuronal cell death [41]. Because Aβ can disrupt mitochondrial dynamics and modulate amyloidogenic pathways during AD progression, antioxidants have received significant attention as promising agents for dementia treatment.

The efficacy of IOE against glutamate excitotoxicity was reported by Kwon and Lee [30]. IOE significantly attenuated glutamate-induced ROS production and inhibited apoptosis in HT22 hippocampal neurons Notably, heme oxygenase-1 (HO-1), a stress-inducible enzyme that provides antioxidant defense to neurons, has been identified as a molecular target of IOE. Glutamate-induced HO-1 downregulation was restored by IOE, and the MAPKs/Nrf2 signaling pathway was identified as the intracellular cascade responsible for mediating this protective effect (Figure 1B).

These findings are further supported by an animal model of glutamate excitotoxicity. In trimethyltin (TMT)-treated mice, oral IOE administration significantly restored HO-1 expression, normalized MAPKs/Nrf2 signaling to near-control levels, and improved both short- and long-term memory performance (Table 1). These results indicated that IOE effectively mitigated dementia-like symptoms in a mouse model [30].

In normal physiology, ROS, reactive nitrogen species, and other unstable metabolites are continuously generated but maintained at low levels through endogenous antioxidant defense systems [46]. HO-1 degrades heme into bile pigments with strong antioxidant properties, and is typically expressed in response to cellular stress [47]. Notably, under toxic conditions induced by excessive glutamate, HO-1 expression is downregulated, which may exacerbate oxidative stress in neurons [30]. HO-1 expression restoration by IOE suggests that rebalancing the cellular oxidative status contributes to the therapeutic effects of IOE on glutamate excitotoxicity. In addition, the specific intracellular signaling pathways (MAPKs/Nrf2) connected with IOE-mediated HO-1 expression were verified using in vitro and in vivo models of glutamate excitotoxicity. These results can be utilized to elucidate the molecular mechanisms required for the development of IOE-based anti-dementia therapeutics.

Although further studies are needed to clarify the effects of IOE on NMDAR activation and Ca^2+^ influx, existing evidence demonstrates that IOE attenuates the key pathological features of glutamate excitotoxicity, including neuronal death and memory deficits, in both in vitro and in vivo models. These results suggest that IOE has a strong potential as a therapeutic candidate for dementia associated with glutamate excitotoxicity.

## 4. Bacterial-Driven Neuroinflammation Attenuation by *I. okamurae* by Targeting the TLR-4/MyD88 Pathway

Neuroinflammation is an inflammatory response that occurs in the central nervous system (CNS). Under normal conditions, inflammation plays a protective role by activating the immune system, eliminating toxic debris, and promoting tissue repair [48]. However, chronic or severe inflammation induced by viral or bacterial infections, toxic metabolites, or traumatic brain injury can contribute to neuronal damage and lead to neurodegenerative diseases, such as AD and PD [49]. Consequently, neuroinflammation has received considerable attention as a central mediator of neurodegenerative disease progression.

Among the various causes of neuroinflammation, bacteria-driven neuroinflammation has recently been considered as an important therapeutic target for dementia treatment. Neuroinflammation is closely associated with systemic inflammation such as infection, periodontitis, and sepsis, suggesting that bacteria or their derivatives can invade the brain via peripheral inflammatory lesions [50,51]. Certain bacterial strains isolated from nasal or pulmonary inflammation sites have been reported to invade the brain [52]. Indeed, 5–10 times more bacteria have been detected in the brains of patients with AD than in healthy controls, and bacterial composition differs significantly between AD and non-AD brains [53]. Furthermore, LPS, a byproduct of Gram-negative bacteria, can penetrate the BBB and promote neuroinflammation [54]. Thus, increased bacterial burden in the body may represent a considerable risk factor for dementia.

The expansion of the pro-inflammatory microbiota in the gut is closely associated with the neurodegenerative disease progression [55]. Although the exact mediators of the gut–brain axis remain to be fully elucidated, pathogenic gut microbiota-derived LPS has been proposed as a potential driver of neuroinflammation [56]. Therefore, restoring the gut microbiota balance in patients with dementia using natural agents or probiotics could represent an effective strategy for improving physiological outcomes and cognitive dysfunction.

Bacteria-mediated inflammatory responses in the brain are primarily initiated in microglia and astrocytes through the activation of TLRs [57,58]. Upon stimulation by pathogens or LPS, TLR-mediated signaling triggers pro-inflammatory cytokine production, ultimately leading to neuronal dysfunction [59]. Thus, each step in the inflammatory cascade induced by bacteria or their derivatives represents a potential therapeutic target for the prevention of bacteria-driven neuroinflammation and dementia progression.

Various natural agents exhibit anti-neuroinflammatory activities [60,61]. Their efficacy has been evaluated in inflammatory neuronal cell systems and some candidates have been tested in neuroinflammatory animal models. However, for clinical development, it is essential to demonstrate their efficacy in both in vitro and in vivo models and to elucidate the molecular mechanisms by which they attenuate bacteria-driven inflammatory responses.

The anti-inflammatory efficacy of IOE has been evaluated in a previous study [31]. Oral IOE administration to mice subjected to LPS-induced neuroinflammation via intracerebroventricular (ICV) injection significantly improved both short- and long-term memory deficits. Notably, IOE administration restored TLR-4 and MyD88 expression in the brain of LPS-injected mice, indicating that IOE effectively modulated key inflammatory signaling molecules in vivo. Additionally, the anti-neuroinflammatory activity of IOE was confirmed in an inflammatory glioma cell model. IOE clearly attenuated LPS-mediated TLR-4 activation and suppressed LPS-induced production of nitric oxide and pro-inflammatory cytokines, including interleukin (IL)-6 and IL-8 (Table 1 and Figure 1C).

TLR-4, a pattern recognition receptor located on the cell surface, is a molecular target of IOE for attenuating bacteria-induced neuroinflammation. Moreover, the involvement of the TLR-4/myeloid differentiation MyD88-dependent signaling pathway was clearly demonstrated in both neuronal cells and the mouse brain. These findings strongly suggest that IOE modulates the signaling cascades triggered by diverse pathogen-associated molecular patterns (PAMPs).

Since TLR-4 is a potent therapeutic target not only for neuroinflammation but also for various immunological disorders, IOE has great potential that IOE could effectively modulate the pathogenesis of autoimmune diseases [62]. In addition, suppression of TLR-4 signaling in Aβ-induced AD models restores cognitive impairments, reduces Aβ accumulation, and prevents neuronal death [63,64]. Taken together, these results strongly suggest that IOE has the potential to attenuate the progression of dementia, particularly transduced through TLR-4/MyD88 dependent pathways. Moreover, IOE oral administration not only reduced neuroinflammatory responses but also improved LPS-induced cognitive dysfunction in mice, highlighting its therapeutic potential as an edible natural agent for dementia patients with bacterial-driven neuroinflammation.

## 5. Marine Algae-Derived Anti-Dementia Agents

Several natural agents derived from marine algae possess anti-dementia properties (Table 2). Among them, extracts from *Ecklonia cava* have shown notable anti-AD and anti-neuroinflammatory activity. Oral administration of a 70% ethanol extract of *E. cava* improves cognitive function by enhancing synaptic activity in an AD mouse model [65]. Moreover, the 50% ethanol extract of *E. cava* demonstrated antioxidant efficacy in H_2_O_2_-treated PC12 neuronal cells [66]. In addition, oral administration of *E. cava* water extract attenuated LPS-induced neuroinflammation and neuronal death [67]. Collectively, these independent studies indicate that *E. cava* exhibits promising anti-dementia potential. However, standardization of E. cava-derived agents is necessary to strengthen their therapeutic efficacy, as each study utilized extracts pre-pared using different solvents and methods.

The anti-AD effects of *Sargassum fusiforme* have been demonstrated using a chronic AD model mouse (APPswe/PS1ΔE9) [68]. Oral administration of chloroform and methanol (2:1) extracts of *S. fusiforme* improved memory function and reduced Aβ plaque formation in the mouse brain. Liver X receptor β (LXRβ) has been suggested as a potential molecular target of *S. fusiforme*; however, the precise mechanism by which *S. fusiforme* activates LXRβ remains to be elucidated.

*Sargassum horneri* is another marine-derived candidate with anti-dementia potential. Limited evidence has demonstrated that the CH_2_Cl_2_-soluble fraction of *S. horneri* exerts protective effects in cellular models of neuroinflammation and gluta-mate excitotoxicity [69]. Treatment with the CH_2_Cl_2_-soluble fraction of *S. horneri* suppressed inflammatory mediator expression, such as tumor necrosis factor (TNF)-α and IL-6 in BV2 microglial cells. In addition, glutamate-induced ROS overproduction was ameliorated in the same fraction in HT22 cells.

Another study further supported the anti-neuroinflammatory activity of *S. horneri*, showing that a 70% ethanol extract produced similar inhibitory effects on LPS-induced neuroinflammation in BV2 cells [70]. Although reductions in activated astrocytes and microglia have been observed in the brains of mice orally administered 70% ethanol extracts of *S. horneri*, the detailed physiological roles of these extracts in modulating neuroinflammation and cognitive impairment remain unclear.

Since the active ingredients can vary depending on the extraction method used to prepare the natural agents, it is generally recognized that extracts produced by different methods may be regarded as distinct natural agents, even if derived from the same species. Therefore, the standardization of extraction protocols is a prerequisite for the development of natural agent–based therapeutics. Although several marine-derived natural agents have demonstrated anti-dementia properties, further experimental evidence is required to establish their potential as multitarget anti-dementia agents.

The IOE was prepared using 70% ethanol and evaluated in three different dementia models. Furthermore, the analytical conditions for the identification of its single bioactive component, diphlorethohydroxycarmalol (DPHC), were optimized using high-performance liquid chromatography (HPLC) and mass spectrometry (MS) [29]. Consequently, IOE may be considered the most extensively characterized marine algae-derived material for multitarget anti-dementia applications. The consistent in vitro and in vivo results obtained from the three independent dementia models provide compelling evidence that IOE exhibits superior efficacy in ameliorating dementia progression compared to other marine algal natural agents.

## 6. Limitations

Although the anti-dementia properties of IOE have been evaluated both in vitro and in vivo, clinical and pharmacokinetic evaluations are essential for its development as an anti-dementia therapy. Because IOE is derived from the edible marine resource, *I. okamurae*, its safety profile is expected to be favorable. Moreover, the doses that exhibited anti-dementia efficacy in animal experiments (7.5 and 12.5 mg/kg bodyweight/day) correspond to human equivalent doses of approximately 36.45 mg and 60.75 mg for a 60 kg adult [29], suggesting potential applicability to human use. Although additional studies are needed to determine the minimum effective dose, IOE may offer advantages in toxicological assessments and pharmacokinetic studies, facilitating clinical translation. Further clinical trials are warranted to confirm the efficacy of IOE in patients with dementia.

Furthermore, the specific functional constituents of IOE responsible for modulating dementia-related symptoms have not been fully identified. Among the known single components, DPHC, a 124 kDa phlorotannin, has been characterized as an analytical marker compound for IOE [29] and has demonstrated neuroprotective activity in H_2_O_2_-treated neuronal cells [71]. Therefore, DPHC may be a key bioactive component contributing to the anti-dementia effects of IOE. However, comprehensive profiling of the individual constituents of IOE, followed by functional validation through in vivo studies, is essential to advancing IOE the pharmaceutical development of IOE.

## 7. Conclusions and Prospects

In this review, the anti-dementia properties of IOE are highlighted and compared with those of other natural agents derived from marine algae. The anti-dementia efficacy of IOE has been demonstrated in three dementia models: AD, glutamate excitotoxicity, and bacterial-driven neuroinflammation. Furthermore, the target molecules and modulatory mechanisms by which IOE regulates the progression of each disease have been elucidated. Collectively, these findings indicate the strong potential of IOE as a natural multitarget agent for dementia therapy.

Standardization through comprehensive chemical characterization is essential for the clinical evaluation of IOE. In addition, large-scale production of IOE under current good manufacturing practice (cGMP) conditions must be optimized to ensure reproducibility and a consistent composition of analytical marker compounds. If positive results are obtained in clinical trials, IOE could be developed as an effective novel therapeutic agent capable of simultaneously modulating multiple pathogenic factors involved in dementia.

## Figures and Tables

**Table 1:** Effects of IOE on the three different disease models of dementia.

Disease Models		Extract	Animal/Cell	Constructs	IOE Treatments	Effects	Ref.
Alzheimer’s disease	In vivo	70% EtOH	C57BL/6 male mice (4-weeks-old)	Aβ_25–35_ ICV-injection (410 μmol/5 μL)	Oral gavage for 4 weeks (7.5, 12.5 mg/kg bw/day)	- Memory deficits [↓], - Aβ plaque [↓] - Neuronal loss [↓], - Apoptosis [↓], - Neuroinflammation [↓]	[29]
	In vitro		Rat pheochromocytoma cells (PC12)	Aβ_25–35_ (70 µM) for 14 h	Pretreated for 2 h (0.075, 0.125 mg/mL)	- Apoptosis [↓], - Neurotoxicity [↓] - Neuroinflammation [↓] - Oxidative stress [↓]	
Glutamate excitotoxicity	In vivo	70% EtOH	C57BL/6 male mice (4-weeks-old)	TMT IP-injection (2.5 mg/kg/bw)	Oral gavage for 3 weeks (20 mg/kg bw/day)	- Memory deficits [↓] - Neuronal loss [↓], -Apoptosis [↓] - Oxidative stress defense system [↑]	[30]
	In vitro		Mouse hippocampalneuronal cells (HT22)	Glutamate (5 mM) for 16 h	Pretreated for 2 h (0.05, 0.1 mg/mL)	- Apoptosis [↓], -Neurotoxicity [↓] - Oxidative stress defense system [↑]	
Bacterial-driven neuroinflam-mation	In vivo	70% EtOH	C57BL/6 male mice (4-weeks-old)	LPS ICV-Injection (4 μg/μL, 3 μL)	Oral gavage for 4 weeks (20 mg/kg bw/day)	- Memory deficits [↓], -Aβ plaque [↓] - Neuronal loss [↓], - Neuroinflammation [↓] - Activation of TLR-4 signaling [↓]	[31]
	In vitro		Rat glioblastoma multiforme cells (C6 glioma)	LPS (1 µg/mL) for 15 h	Pretreated for 2 h (0.1, 0.2 mg/mL)	- Activation of TLR-4 signaling [↓] - Neuroinflammation [↓] - Oxidative stress [↓]	

↑: upregulate, ↓: downregulate.

**Table 2:** Anti-dementia properties of natural agents derived from marine algae.

Species	Extracts	Neurotoxic Agents	Experimental Models	Study Type	Effects	Refs.
*Ecklonia cava*	70% EtOH	Aβ_1–42_	ICR mice (410 μmol/10 μL, ICV injection)	In vivo	- Attenuation of cognitive deficits - Reduction in oxidative stress - Improvement of mitochondrial dysfunction - Enhancing the synapse function	[65]
	50% EtOH	H_2_O_2_	PC12 cells, SH-SY5Y cells	In vitro	- Reduction in oxidative stress	[66]
	Water	LPS	ICR mice (750 μg/kg/bw, 1.5 mg/kg/bw)	In vivo	- Inhibition of neuroinflammation - Attenuation of neuronal apoptosis	[67]
*Sargassum fusiforme*	Chloroform and methanol (2:1 (*v*/*v*))	-	APPswePS1ΔE9 mice	In vivo	- Improvement of memory function - Attenuation of Aβ plaque formation	[68]
*Sargassum horneri*	CH_2_Cl_2_ -soluble fraction of 70% EtOH extracts	LPS and glutamate	BV2 cells HT22 cells	In vitro	- Inhibition of neuroinflammation - Attenuation of cell damage - Reduction in oxidative stress	[69]
	70% EtOH	LPS	BV2 cells C57/BL6 mice (1 mg/kg, IP injection)	In vitro and In vivo	- Attenuation of inflammation - Inhibition of microglial activation - Modulation of p38MAPK/NF-κB pathway	[70]

*E. cava*, *S. fusiforme*, and *S. horneri* were selected through searches in PubMed and Google Scholar using single or combined keywords (seaweeds, algae, AD, glutamate, LPS, and H_2_O_2_).

## Data Availability

The raw data supporting the conclusions of this article will be made available by the authors on request.
